# Biophysical models of early mammalian embryogenesis

**DOI:** 10.1016/j.stemcr.2022.11.021

**Published:** 2023-01-10

**Authors:** Alaina Cockerell, Liam Wright, Anish Dattani, Ge Guo, Austin Smith, Krasimira Tsaneva-Atanasova, David M. Richards

**Affiliations:** 1 Living Systems Institute, University of Exeter, Stocker Road, Exeter EX4 4QD, UK; 2 Department of Mathematics, University of Exeter, North Park Road, Exeter EX4 4QF, UK; 3 EPSRC Hub for Quantitative Modelling in Healthcare, University of Exeter, Exeter EX4 4QJ, UK; 4 Department of Bioinformatics and Mathematical Modelling, Institute of Biophysics and Biomedical Engineering, Bulgarian Academy of Sciences, 105 Acad. G. Bonchev Street, 1113 Sofia, Bulgaria; 5 Department of Physics and Astronomy, University of Exeter, North Park Road, Exeter EX4 4QL, UK

**Keywords:** biophysical modeling, computational simulation, embryogenesis, blastocyst, blastoid

## Abstract

Embryo development is a critical and fascinating stage in the life cycle of many organisms. Despite decades of research, the earliest stages of mammalian embryogenesis are still poorly understood, caused by a scarcity of high-resolution spatial and temporal data, the use of only a few model organisms, and a paucity of truly multidisciplinary approaches that combine biological research with biophysical modeling and computational simulation. Here, we explain the theoretical frameworks and biophysical processes that are best suited to modeling the early mammalian embryo, review a comprehensive list of previous models, and discuss the most promising avenues for future work.

## Graphical abstract

**Figure:**
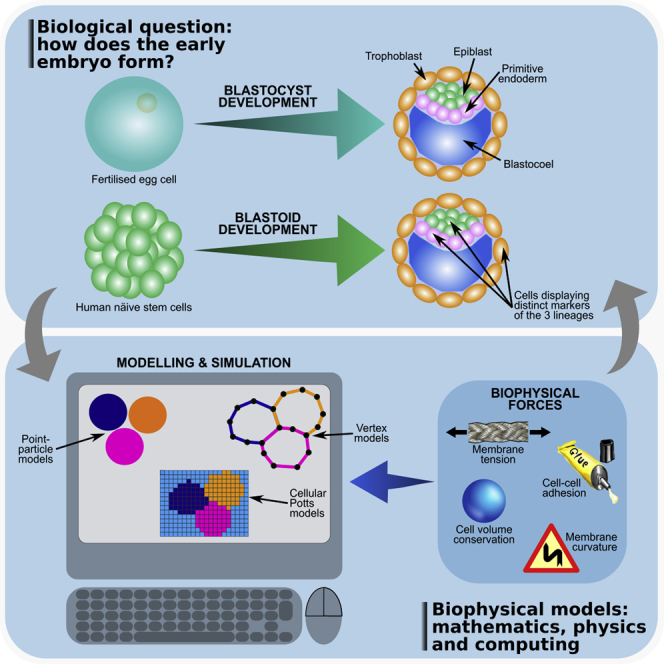

## Teaser

In this article, Richards and colleagues review the theoretical frameworks and biophysical processes that are best suited to modeling development of the early mammalian embryo. They discuss previous work in this area, show preliminary results from a new cellular Potts model, and explain how novel stem cell systems called blastoids are starting to help design, fit, and validate biophysical models.

## Introduction

**Figure 1:**
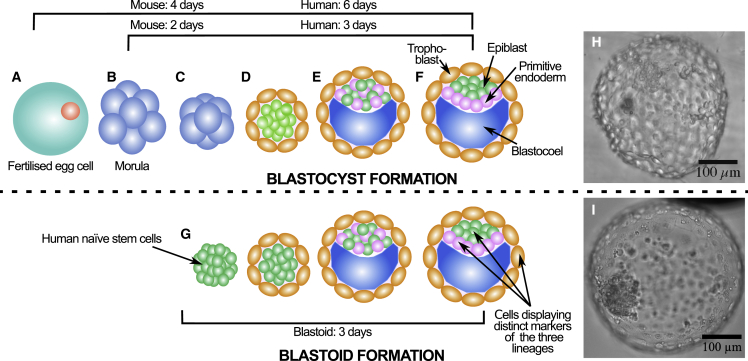
Schematic representation of blastocyst (top row) and blastoid (bottom row) development (A–G) The fertilized egg cell (A), the morula (formed after the egg undergoes several cleavage divisions) (B), the compacted morula (with increased cell contact area) (C), polarization and the first lineage specification (leading to a shell of trophectoderm surrounding the inner cell mass) (D), blastocoel formation and inner cell mass differentiation (into epiblast and primitive endoderm cells in a salt-and-pepper pattern) (E), the final mature blastocyst (after the epiblast and primitive endoderm have undergone cell sorting) (F), and blastoid development whereby a clump of naive epiblast cells develop into a blastocyst-like structure and cells display exclusive lineage markers of the three tissue layers (G). (H) Bright-field image of human late blastocyst (E7). Image kindly provided by Jennifer Nichols (University of Edinburgh, UK). (I) Bright-field image of a D3 blastoid formed according to the protocol of Yanagida et al. (2021) (Gerri et al., 2020; Yanagida et al., 2021; Kagawa et al., 2021). Scale bars: 100 μm.

Formation of the mammalian embryo can be divided into a number of stages, with the earliest covering development from a zygote to the first couple of hundred cells arranged in a structure termed the blastocyst. The blastocyst, shown in Figure 1, consists of an epithelial layer of cell (trophectoderm) encasing a fluid-filled blastocoel and the inner cell mass (ICM) (epiblast and primitive endoderm cells). This earliest stage, which will be the exclusive focus of this review, is particularly fascinating since it is unique to mammals, is still incompletely understood, lays the basis for all subsequent development, and involves some of the most fundamental processes in embryogenesis, including the first lineage specification and establishment of the initial polarity axis (Beddington and Robertson, 1999; Gerri et al., 2020).

This first stage also shows fascinating variation between species, with some processes (such as polarization and cell communication through tight junctions) being highly conserved, while others (such as the timeline of development and cell specification mechanisms) show significant differences (Gerri et al., 2020; Tocci, 2020). Why some mechanisms are so well conserved across species while others are not is not well understood and requires further study of not just human embryogenesis but other mammals as well (Frankenberg et al., 2016).

Development of the blastocyst is not only of fundamental interest but also has great significance for a number of globally important issues, including human fertility, improving assisted human conception (for example, IVF where the majority of human embryos fail to develop beyond implantation), conservation efforts (such as aiding fertility of endangered species), and global food security (via economically important animals such as pigs, cows, and sheep) (Wildt et al., 1992; Achache and Revel, 2006; Mutembei et al., 2015; Hernandez Gifford and Gifford, 2013).

Modern research in the life sciences increasingly applies multidisciplinary approaches, where traditional experimental work is closely combined with theoretical techniques such as biophysical modeling and computer simulation (Montes-Olivas et al., 2019). Nowhere is this more true than in embryogenesis, with a number of mathematical models of blastocyst formation appearing in recent years (Krupinski et al., 2011; Cang et al., 2021; Maître et al., 2016; Dumortier et al., 2019; Nissen et al., 2017; Honda et al., 2008; Giammona and Campàs, 2021; Le Guillou et al., 2009; Yanagida et al., 2022).

Biological systems are typically complex, often making it difficult to determine the governing principles. Modeling seeks to simplify a system so that the key underlying behavior can be elucidated and investigated, making hypothesis testing easier. A good model (which should always be driven and informed by experimental data) should be able to make experimentally testable predictions and suggest future experiments, reducing the need for tricky, expensive, or time-consuming lab work (Montes-Olivas et al., 2019). Even models that fail can help indicate that one or more processes are missing from current knowledge (Krupinski et al., 2012). Modeling can also help explore system robustness and shed light on how and when a system might fail, a topic of particular relevance to the IVF clinic (Firmin and Maître, 2021). Finally, modeling can also help identify common mechanisms and principles that operate across organisms, something that is often difficult to determine from purely experimental approaches (Dahl-Jensen and Grapin-Botton, 2017).

Using experimental data to validate and fit models is a crucial step without which modeling loses all usefulness. Animal models, especially mice, are a common choice for this and play an integral step in building confidence in model predictions (Dumortier et al., 2019; Yanagida et al., 2022; Le Guillou et al., 2009). However, it is important to remember that while blastocysts share broadly similar structures across mammals, there are important underlying differences. For example, gene networks are only partially conserved between species, and the details of the associated signaling pathways often differ (Chazaud and Yamanaka, 2016). This means that caution must be exercised when using a given animal model to draw conclusions about development in other organisms (Gerri et al., 2020; Burton, 1992). Research on the human blastocyst is particularly challenging, not least because of important ethical considerations related to working with human embryos. Stringent regulations are necessary so that research is ceased before the embryo is considered an individual and to aid in ensuring public trust (Matthews et al., 2021).

A new type of experimental model system based on stem cell technology that is likely to provide significant data for future models has recently been developed. These stem cell models, called blastoids, involve differentiating stem cells to create a structure that resembles the blastocyst itself. First investigated in mice (Rivron et al., 2018), there are now also a number of examples of the human blastoid model (Yanagida et al., 2021; Yu et al., 2021; Liu et al., 2021; Kagawa et al., 2021). Blastoids, which are not constrained by the same strict licensing regulations that apply to human embryo research, offer a possible avenue to mitigate some of the ethical issues involved in embryo research and provide substantially higher throughput data (Lovell-Badge et al., 2021).

Human blastoids may closely resemble the human embryo blastocyst in size, morphology, and cellular composition. This is rather remarkable because they form not from unspecialized morula cells as in the embryo but from stem cells with an epiblast identity (Figure 1) (Yanagida et al., 2021). As modeling helps simplify a system to the fundamental ingredients, studying how apparently identical multicellular structures (both in blastocysts and blastoids) form from different starting cell types may shed light on underlying mechanisms and the relative importance of the key factors at play. Even if these processes are different between blastocysts and blastoids, this will have interesting implications for the mechanisms of embryogenesis and the origin of system robustness.

Biophysical modeling and computational simulation, the topic of this review, focus in particular on the physical principles such as forces and interactions that govern the behavior of biological bodies, in this case cells. Such models are particularly useful in morphogenetic contexts including many areas of development, where cell shape, motion, and interaction are of key importance (Fletcher et al., 2014). The biological details of individual genes and proteins can be important to be included, but biophysical considerations (such as membrane tension, cell-cell adhesion, and fluid dynamics), which are affected by these subcellular processes, are highlighted here. Biophysical modeling can be extended to include subcellular processes such as gene regulatory networks (GRNs) and their role in cell specification, but they will not be the main focus of this review (Krupinski et al., 2011; Cang et al., 2021).

The kinds of question that can be addressed regarding biophysical modeling of embryogenesis include the role of surface tension in cell internalization (Giammona and Campàs, 2021; Maître et al., 2016), the relative importance of differential adhesion, apoptosis and noise in cell sorting, the effect of the hard outer shell (the zona pellucida) on cell packing, the origin of system robustness, and the minimum set of ingredients required for successful blastocyst formation (Krupinski et al., 2011).

For example, in the case of IVF, modeling could be useful in determining which physical processes are often implicated in blastocyst failure, allowing laboratories to improve their embryo profile grading system. Since most clinics employ static morphological assessments of embryos, modeling could also investigate the effect of relative cell numbers in the ICM and trophectoderm or the relative cell diameters on blastocyst failure rates (Cimadomo et al., 2022).

This review is organized as follows. After an introduction and overview of blastocyst formation, we discuss possible modeling frameworks suitable for describing early embryogenesis, including cell-density approaches, cellular Potts, vertex models, level-set, phase field, subcellular element modeling, and the finite element method. Next, we introduce the various relevant topics from physics, focusing on the key cellular and intercellular forces that must be considered when designing biophysical models of the blastocyst. We then cover the various existing models for early mammalian embryogenesis, describing the main results, strengths, and weaknesses of each. Finally, we provide a perspective on the future of the field, suggest the most promising modeling avenues and frameworks, and describe how multidisciplinary approaches will be critical to future progress in this area.

## Overview of early embryo development

Although many aspects of blastocyst development are broadly conserved across mammals, there are important differences between species (Gerri et al., 2020). So far mouse models have been instrumental in the development of this area and are by far the most common model used in blastocyst research. Therefore, we will use the mouse as a reference in this section while pointing out differences with humans where appropriate (Cockburn and Rossant, 2010). While key genes and gene networks are not the focus of this review, some details are included here to aid understanding of the models discussed later.

The blastocyst consists of an outer trophectoderm layer (which later develops into the placenta), the inner cell mass (ICM), and a fluid-filled cavity called the blastocoel (Figure 1). The ICM itself partitions into two lineages: the epiblast, which will give rise to the embryo proper, and the primitive endoderm, which will form the yolk sac. The primitive endoderm is also called the hypoblast. At the same time, the trophoblast matures to mediate implantation into the uterus (Cockburn and Rossant, 2010; Herrera-Delgado and Maître, 2021).

Embryogenesis is initiated by fusion of the egg and sperm. The fertilized egg first undergoes several rounds of cleavage into cells called blastomeres. Unlike regular cell division, cleavage is not associated with subsequent cell growth, so that each cleavage event produces smaller and smaller cells with the total cell mass and volume approximately that of the original egg (Gerri et al., 2020). The mass of cells, which at this stage is referred to as a morula, are surrounded by the zona pellucida (ZP), a sugar layer that places constraints on blastomere positioning (Fierro-González et al., 2013; Maître, 2017).

At the 8-cell stage in mice the cells undergo compaction, whereby cells adhere together and the total surface area of the blastocyst is reduced. There is still no consensus on the origin of this process. Fierro-González et al. (2013) claim that this is driven by cadherin-dependent filopodia that form between the 8- and 16-cell stage. These protrusions undergo tension forces that allow cells to flatten against each other (Lim and Plachta, 2021). However, it has been proposed more recently that compaction occurs instead through an increase in tension at the free cell surface, mediated through an increase in actomyosin levels that causes the free surface to contract (Maître, 2017; Heisenberg and Bellaïche, 2013). In this proposed mechanism, the filopodia are thought to prevent contractility increasing at cell-cell interfaces, with the increased surface tension at the free surface acting to reduce the cell-medium surface area and the reduced surface tension of cell-cell contacts acting to increase contact surface area (Maître, 2017). It is not clear which mechanisms for compaction are conserved between mice and humans, although E-cadherin expression has been observed during human compaction (Iwata et al., 2014), suggesting a similar role for cadherin-dependent filopodia (Tocci, 2020).

The 8-cell stage is also when polarization occurs (Chazaud and Yamanaka, 2016). During this process, apical domains form at the free surface of the cells, starting from the center and expanding outward to eventually meet at cell junctions and anchoring neighboring cells (Lim and Plachta, 2021). These apical domains are thought to be the source of the difference in surface tension between internalizing and non-internalizing cells (Maître et al., 2016; Maître, 2017) and are also believed to be linked to cell fate specification (Maître, 2017; Zhu et al., 2021b). The apical domain is conserved through asymmetric division where one daughter cell is external (with an apical domain) and the other is internal (without an apical domain). However, cells can also undergo symmetric division (whereby the daughter cells share the same properties). Other mechanisms, such as polarity, are also believed to be able to control cell division: the apical domain, for example, is known to orient the division plane by influencing spindle position (Maître, 2017; Lim and Plachta, 2021; Gillies and Cabernard, 2011). Although an apical region that differs from the basolateral region of the cell has been observed in humans, there is still limited research into the apical domains of human blastocysts (Tocci, 2020).

A key process is cell internalization, whereby cells transition from having contact with the outside medium to being entirely inside the cell cluster. This can be achieved, for example, through oriented division. However, apolar cells at the cell-medium boundary have also been observed to internalize (Chazaud and Yamanaka, 2016; Maître, 2017; Lim and Plachta, 2021). Samarage et al. proposed that apical constriction, driven by circumferential contractile networks, is the chief driving force behind internalization. Apical constriction is a ubiquitous morphogenic process mediated through adhesion and cortical tension (Samarage et al., 2015; Martin and Goldstein, 2014). However, Maître et al. (2016) suggested that the reduced myosin levels in the apical domain of polarized cells lower their contractility, causing apolar cells to internalize. The processes of compaction, polarization, and internalization have been predominantly studied in mice, so it is unclear how much is conserved in humans. Morphologically, mouse and human development appears to be similar at least on a macroscopic level, although there are differences in terms of timing (Cockburn and Rossant, 2010).

The next key event is the first lineage specification, which results in an outer layer of trophectoderm, an epithelial layer held together with tight junctions that surrounds the ICM (Chazaud and Yamanaka, 2016; Maître, 2017). The key transcriptional regulators involved in mouse embryos are Cdx2 for trophectoderm and Oct4 for ICM cells, along with a number of identified signaling pathways such as Hippo (Wu and Schöler, 2014; Pesce and Schöler, 2001). The Hippo pathway inactivates YAP (a transcriptional co-activator) in ICM cells so that YAP only localizes in the nuclei of outer cells. This YAP localization is integral for trophectoderm lineage specification in mice, although its role has not been fully explored in humans (Rayon et al., 2014; Tocci, 2020). It is currently thought that cell polarization (and, hence, cell position) controls which cells end up in trophectoderm and ICM fates (Tarkowski and Wróblewska, 1967; Johnson and Ziomek, 1981). The increased number of cell contacts of the inner cells reduces their contractility, with reduced cell contractility then being associated with inner cell fate (Maître, 2017; Maître et al., 2016; Maître et al., 2015). The factors that influence the first specification process are still poorly understood, and further research in this area is likely to benefit from the biophysical approach we describe here (Lim and Plachta, 2021; Arroyo and Trepat, 2019).

Blastocoel development begins from the late 32-cell stage. Actin rings in the apical domains of the trophectoderm cells expand and anchor cells at their junctions. The trophectoderm cells express ion pumps which then create an osmotic gradient, resulting in a fluid flux across the permeability barrier of the cells into the apical compartment of the blastocyst (Dumortier et al., 2019; Lim and Plachta, 2021; Eckert and Fleming, 2008; Le Verge-Serandour and Turlier, 2021). Recently it has been argued that, rather than the blastocoel forming from one location, many cell-cell contacts are fractured forming multiple dispersed microlumens (Dumortier et al., 2019). These microlumens then coalesce, due to pressure differences that can be described by the Young-Laplace equation. This mechanism may also be able to explain the positioning of the blastocoel between the trophectoderm and ICM cells without any geometric influence from the ZP. Finally, the blastocoel is believed to go through cycles of growth and collapse before stabilizing (Lim and Plachta, 2021). Microlumens and hydraulic fracturing in humans has yet to be fully assessed and will be an interesting focal point for further research (Gerri et al., 2020).

The final developmentally relevant event here is the specification and sorting of the epiblast and primitive endoderm cells. The lineage-associated markers for primitive endoderm cells (Gata6) and epiblast cells (Nanog) are originally co-expressed in the ICM. The divergence of the two lineages is regulated in mice through fibroblast growth factor (FGF) signaling, although this may differ in humans (Gerri et al., 2020; Kuijk et al., 2012). These cells are initially found in a “salt-and-pepper” pattern (Bessonnard et al., 2014) before undergoing sorting so that the primitive endoderm cells coat the blastocoelic surface of the ICM (Chazaud and Yamanaka, 2016). While OCT4, Nanog, Gata6, and several other key epiblast and primitive endoderm transcription factors are conserved between mouse and human, there are also significant differences (Boroviak et al., 2018). Further work is needed to fully understand and appreciate these differences and how they affect lineage specification.

The mechanisms behind epiblast and primitive endoderm sorting are still not fully known. Currently they are thought to involve differing cellular physical properties (e.g., adhesion, contractility, or surface fluctuations) as well as a role for apoptosis of misplaced cells (Maître, 2017; Krupinski et al., 2011; Yanagida et al., 2022). The differential adhesion hypothesis suggests that adhesion is the dominant factor determining surface tension between neighboring cells and that minimization of surface energy is the root cause of cell sorting (Durand, 2021; Lecuit and Lenne, 2007). However, it has also been argued that differential adhesion alone cannot produce the required tension (Brodland and Chen, 2000). Yanagida et al. (2022) found that the E-cadherin adhesion molecules were not differentially expressed at the protein level between primitive endoderm and epiblast cells and instead suggested that differential cell surface fluctuations lead to differing cell fluidity and, hence, cell sorting.

## Possible modeling approaches

This section discusses the possible frameworks for modeling mammalian blastocyst development, covering both existing models and other frameworks that we believe will be useful in the future. We only consider models that operate at the length scale of cells and above. This is not to say that intracellular processes (such as the interactions between individual proteins and genes) are not important, nor that such processes are not amenable to modeling, simply that we focus on questions concerning overall embryo layout, morphology, and quality, especially those driven by biophysical considerations. As such, in this review we mainly focus on physical effects (such as forces and energy) rather than intracellular signaling details (such as GRNs). However, we do, when appropriate, discuss the adaptation of these models to include subcellular processes.

The differences between species mean that a single model is unlikely to work for all mammals. However, we do expect the relevant underlying biophysical forces to remain the same, even if their origins and magnitudes differ. This means that a given modeling framework may well apply across mammals, but with different parameters for each organism (Gerri et al., 2020).

It is beyond the scope of this review to include all possible modeling approaches. We have instead chosen to focus on those approaches that describe embryogenesis at the cellular level, either as individual cells or in a coarse-grained representation. Other model frameworks that are appropriate to later stages of morphogenesis (where cell numbers are higher), such as the self-propelled Voronoi model for densely packed epithelial cells (where cells are considered as point particles but their energy functional is dependent on cell geometry) (Ai et al., 2022; Bi et al., 2015, 2016), are not covered.

Although individual models rarely fall into neat categories, it is often helpful to classify modeling frameworks into distinct types so that different approaches can be compared and contrasted. For example, models can either describe the full three-dimensional (3D) embryo or focus on only a two-dimensional (2D) cross-section or projection. Furthermore, models can be theoretical or phenomenological, or deterministic or stochastic.

**Figure 2:**
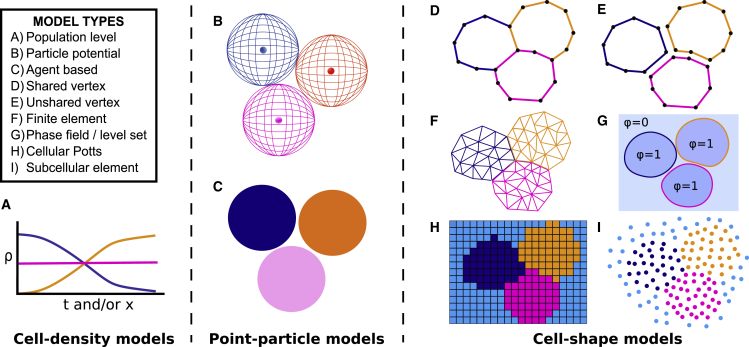
Possible model frameworks for simulating blastocyst development (A) Cell-density models: ordinary differential equations or partial differential equations are used to describe the cellular density *ρ*(*x*,*t*) as a function of position *x* and/or time *t*. (B and C) Point-particle models: cells are treated as point-like and interact either via a given potential (B) or via a set of agent-based rules (C). (D–I) Cell-shape models: the full cell shape is described. These include vertex models that discretize the cell membrane as a set of connected points (with neighboring cells either sharing vertices [D] or consisting of separate vertices [E]), finite element models where the cell is discretized into simpler subdomains such as triangles (F), the phase-field method (with the field *φ* = 1 inside the cell and *φ* = 0 outside) and level-set formalisms that describe cells via deforming contours and moving boundaries (G), cellular Potts models where cells are represented by multiple lattice points (H), and subcellular element models where the cell is discretized as interacting points (I).

Another important model classification is the level at which cells are described. As shown in Figure 2, there are broadly three ways this can be done: cell-density models (which do not consider individual cells), point-particle models (where cells are described as unstructured points or incompressible point-like objects), and cell-shape models (which consider the full cell shape). We now consider each of these in turn.

### Cell-density models

Cell-density models do not describe individual cells but focus on the cellular density *ρ*(*x*,*t*) at position *x* and time *t* (Figure 2A). Different cell types can be included via different fields with, for example, epiblast and primitive endoderm cells each having their own densities. This approach naturally lends itself to a system of differential equations (typically partial differential equations), with interaction terms describing how cell types influence each other (Maini et al., 1999; Chaturvedi et al., 2005). These models have the advantage of being relatively simple and being amenable to the wealth of existing analysis methods. However, they struggle to capture details about individual cells and are hence more suitable for systems with far greater numbers of cells than the blastocyst (Boromand et al., 2018).

### Point-particle models

The next level of detail is described by point-particle models, where individual cells are considered, but only as structureless points or incompressible point-like objects. Properties such as cell volume can be included, but more fine-grained details, such as cell deformation, are typically missing. Agent-based modeling naturally lends itself to this type of model, where cells (the agents) move and interact according to (generally probability-based) predefined rules that emulate cellular behavior such as apoptosis of misplaced cells or increased adhesion between cells of the same type (Nissen et al., 2017) (Figure 2C). These models possess a lot of flexibility because agent behavior, agent morphology, and interactions between agents can all be easily incorporated (Glen et al., 2019). Compared with other modeling frameworks, agent-based models are typically computationally inexpensive and can readily capture system heterogeneity, but are typically unable to represent details of cell deformation.

Another example of this type is particle-potential models. Cells are treated as particle-like and interactions are implemented through soft sphere potentials instead of probability-based rules as with agent-based models (Figure 2B). Examples of the potentials used are the Johnson-Kendall-Roberts model and the dashpot-spring elements model (Drasdo and Höhme, 2005; Drasdo et al., 2007; Palsson, 2001). In these models, an implied cell shape can be found as a Voronoi diagram (Okabe et al., 2009). Particle-potential models are typically used for studying cell positioning, or tissue deformation under certain conditions or confinements (Krupinski et al., 2011; Giammona and Campàs, 2021; Nielsen et al., 2020). Although quick to simulate, these models cannot explicitly represent complex cell deformations (Tanaka, 2015). However, they can capture their effects. For example, Nielson et al. implemented a model where cells are treated as point particles that have a polarity axis. Modifying cell-cell forces to favor a tilt in apical-basal polarities allowed them to mimic cell wedging (Nielsen et al., 2020; Nissen et al., 2018).

### Cell-shape models

The final and most detailed level of model framework are cell-shape models, where the precise cell shape is studied. These are by far the most common and arguably the most useful type of modeling employed to describe early mammalian embryogenesis at and before the blastocyst stage. Such models include cellular Potts models, the subcellular element method, finite element models, level-set approaches, phase-field models, and vertex models, each of which we now describe in more detail. Such models have many advantages although, owing to increasing computational complexity, including factors such as system heterogeneity can be more difficult (although by no means impossible) than with point-particle models.

The cellular Potts model is a lattice-based approach that minimizes the Hamiltonian expression of the system energy through a Metropolis-like algorithm (Marée et al., 2007; Graner and Glazier, 1992), with each lattice point identified with either a particular cell or the surrounding medium (Figure 2F). This Hamiltonian is typically considered to be phenomenological and is thus sometimes criticized as non-physical. However, attempts have been made to map the real biophysical properties onto different terms of the model Hamiltonian (Rens and Edelstein-Keshet, 2019; Magno et al., 2015). At each time step the system Hamiltonian is used to determine whether a given lattice point should maintain its identification or be assigned to another cell (Scianna and Preziosi, 2012). The Hamiltonian typically includes an area/volume constraint (for 2D and 3D cells, respectively) as well as an adhesion term between neighboring cells (Graner and Glazier, 1992). One advantage of this type of modeling is that membrane fluctuations are naturally included (Voss-Böhme, 2012).

**Figure 3:**
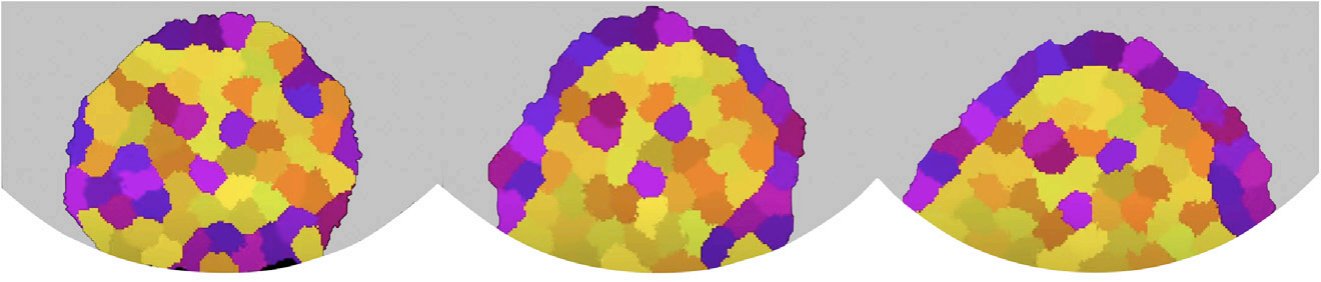
Prototype of a cellular Potts model showing cell sorting of the inner cell mass Three time steps (increasing time from left to right) are shown. Primitive endoderm cells (purple) and epiblast cells (yellow) compose 30% and 70% of the cells, respectively. Gray represents the rest of the blastocyst (including the blastocoel) and white the extracellular medium. Cells are twice as adhesive to other cells of the same type than to cells of the other type. Epiblast cells are twice as adhesive to the cell boundary than primitive endoderm cells are to the boundary. Primitive endoderm cells are approximately three times more adhesive to the medium than epiblast cells are to the medium.

**Figure 4:**
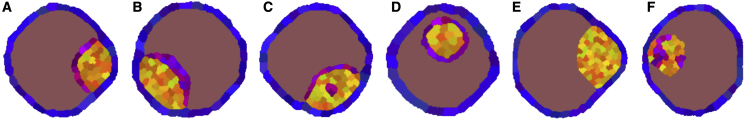
Possible failure mechanisms of inner cell mass sorting suggested by our cellular Potts model Trophectoderm is shown in blue, epiblast in yellow, primitive endoderm in purple, and the blastocoel in brown. Categories: successful blastocyst (A), multilayered primitive endoderm (B), misplaced primitive endoderm cells (C), inner cell mass detached from the trophectoderm (D), complete disappearance of primitive endoderm (E), and failure of primitive endoderm layer formation (F).

Despite its ease of implementation, there are still relatively few models that have used a cellular Potts approach to describe blastocyst formation. The promise in this area is demonstrated by the prototype model shown in Figure 3. The model uses differential adhesion to sort the ICM into the observed pattern of epiblast and primitive endoderm cells. At present, this model does not include apoptosis of misplaced cells or cell fate switching, which would be able to correct misplaced cells in the ICM. However, it is already providing useful results on possible blastocyst failure mechanisms (Figure 4).

In contrast to cellular Potts, the subcellular element method is based on describing the cell as a series of interacting points that are typically not confined to a lattice (Figure 2G). A prescribed potential captures the intra- and intercellular dynamics between these points, which is used to derive a force that describes (typically by assuming overdamped Langevin dynamics) how individual points move (Newman, 2005). For a given point, the potential between other points in the same cell is typically different from that between points in other cells (or the extracellular matrix), which helps ensure that cells stay as contiguous bodies (Tanaka, 2015). The subcellular element is connected to more coarse-grained approaches, such as cell-density models, through mean-field approximations (Newman, 2005).

The finite element method is a type of numerical solution for partial differential equations in which the cell is divided into smaller, simpler subdomains (the elements). For example, the cell could be discretized into a triangular mesh (Figure 2H). Rules then describe how individual elements deform, which are assembled to produce the total deformation of the structure (Bidhendi and Geitmann, 2018; Davidson et al., 1995). These models have often been used to simulate tissue or bone deformations after the blastocyst stage (Allena et al., 2010; Brunt et al., 2015; Chen and Brodland, 2008; Liu et al., 2007). They are also commonly used to address topics in morphogenesis that require continuum mechanics such as, for example, limb development, blastocyst hatching, and embryo invagination (Kida and Morishita, 2018; Tvergaard et al., 2021; Conte et al., 2008). At times, they also have been used at the scale of individual cells (Wu and Pitts, 1999; Mijailovich et al., 2002). While finite element models have many advantages, their computational complexity can be a hindrance when modeling multiple stages of blastocyst development.

Cells can also be represented at the cell-shape level using the level-set formalism, which tracks cell shape via a closed contour that is implicitly defined through a function taking values over the whole domain. Extra- and intracellular forces impact the velocity at which the contour deforms (Yang et al., 2008; Neilson et al., 2011b). Similarly, phase-field approaches can be used on moving interface problems by formulating the free energy of the system in terms of a parameter *φ* that carries a value of 1 inside the cell and zero outside. Free energy minimization and other physical considerations can then be used to generate differential equations that capture how *φ* changes over time (Camley and Rappel, 2017; Moure and Gomez, 2020; Mueller et al., 2019; Zhu et al., 2021a; Najem and Grant, 2016).

The final type of cell-shape model we consider is vertex models. Here the cell membrane (rather than the body of the cell) is discretized and represented by a series of vertices that form a polygon. This is often simpler to implement than the finite element method. Vertices move according to certain rules that can be thought of as implementing the various relevant biophysical forces (such as membrane tension and curvature) (Fletcher et al., 2014). When a vertex model (or finite element method) is used to describe multiple cells (as for the blastocyst), there are two options: either vertices are shared between neighboring cells or each cell is represented by its own set of vertices. In a shared-vertex model (Figure 2D), cells share both vertices and edges and are visually akin to tightly packed epithelial cells, with no gaps between cells. These models can include mechanisms that allow cells to change their connections, enabling phenomena such as cell sorting to occur. Shared-vertex models generally lend themselves to tightly packed epithelial systems found in developmental stages beyond that of the blastocyst (Barton et al., 2017; Alt et al., 2017; Trichas et al., 2012; Spahn and Reuter, 2013), although there has been some work to develop a shared-vertex model framework that can contain gaps between cells (Kim et al., 2021). In non-shared-vertex models (Figure 2E) each cell possesses its own set of vertices, with vertices on neighboring cells able to be bound together. This framework easily allows gaps between cells to form and so, in some ways, is a more suitable framework for modeling compaction and cell sorting (Boromand et al., 2018).

### Inclusion of subcellular processes

This review mainly focuses on models of embryogenesis at the cell level. However, such models can, if necessary, also include subcellular elements such as the cytoskeleton or particular proteins. For example, models including cell specification may include a set of differential equations to capture the role of a key GRN. A few of the models discussed below fall into this category (Krupinski et al., 2011; Cang et al., 2021). For more details, refer to the research by Karlebach and Shamir (2008), Vijesh et al. (2013), and Hasty et al. (2001).

Subcellular phenomena other than GRNs are also included in some models. For example, actin may be of interest for models concerning cell deformation. The role of actin (including polymerization and depolymerization) can be modeled in various ways, including partial differential equations or agent-based modeling. How actin affects cell shape or motion can then be implemented by, for example, cell displacements that are dependent on actin levels (as demonstrated in the finite element model by Hervas-Raluy et al., 2019) or cell markers responding to local actin levels (as in the 3D vertex model by Revell et al., 2019). Additionally, Jamali et al. (2010) developed a subcellular element method for epithelial cells that integrates the viscoelastic nature of the cytoskeleton via Voigt subunits that connect cell membrane elements to nuclear membrane elements.

Another approach is by Neilson et al., who use an evolving surface finite element method that includes activator and inhibitor feedback loops in a model of pseudopod formation in which the perimeter of the cell moves at a velocity normal to the cell boundary and proportional to the local activator level (Neilson et al., 2011a, 2011b). Also, Sego et al. (2020) developed a cellular Potts model of viral immune interactions in epithelial tissues. This model also includes mechanisms for viral internalization of cells from an extracellular virus field, viral replication, viral release, and viral transport, in addition to immune cells, extracellular viruses, cytokines, and an oxidative agent in the extracellular matrix.

### Other considerations

We have described the aforementioned model types mostly in the context of 2D models, where cells are described as 2D regions surrounded by one-dimensional (1D) boundaries representing the cell membrane. However, each framework can also be extended to the more realistic 3D case with a 3D cell body and a 2D membrane. For example, in the finite element method this involves using 3D rather than 2D elements (or volumes), such as tetrahedra rather than triangles. Some processes, such as cell sorting, can be successfully examined in two dimensions (Nissen et al., 2017), whereas others, such as cellular packing, are intrinsically 3D (Krupinski et al., 2011; Le Guillou et al., 2009; Giammona and Campàs, 2021). The relative ease with which each type of model can be extended to three dimensions may be an important factor to consider when choosing a modeling framework.

There are a number of other factors to consider when comparing these modeling frameworks, including biological realism, closeness to real biophysical processes, required level of experimental input, noise, model complexity (along with the number of parameters), ease of model design, and computational resources (run time and memory requirements). Furthermore, there are particular additional factors relevant for blastocyst modeling, including ease of implementing cell division and cell fate specification, whether GRNs can be incorporated (Herrera-Delgado and Maître, 2021), ability to compare to transcriptome data (Cang et al., 2021), and how blastocoel formation and dynamics can be included.

When choosing a modeling framework, the “optimal” choice is not always obvious and often depends on the specifics of the particular process being simulated (e.g., the particular developmental stage being considered) as well as the available data. It is often a trade-off between model complexity, computational expense, and biological realism. It can also be difficult to distinguish what impact the framework itself has on the behavior observed in the simulations. Direct comparison of frameworks, such as the one developed by Osborne et al. (2017), can aid in separating genuine physical phenomena from artifacts of the modeling framework itself (Voss-Böhme, 2012).

## The physics of the blastocyst

Any morphogenetic process requires a combination of biophysical forces working in unison to achieve the desired cell shapes, positions, and dynamics. These include forces attributable to membrane tension, volume conservation, membrane curvature, and intracellular cytoskeletal dynamics. To model these forces in an accurate and effective manner, and to be able to make an informed decision on which forces to include and which to leave out, it is important to first understand their biophysical origin. In this section, we discuss these origins and explain possible model implementations relevant to blastocyst development. For clarity, rather than give expressions for the force itself we give formulae for the associated potential energy, which (if necessary) can be converted to a force by taking the appropriate spatial derivative.

### Membrane and cortical tension

The cell membrane is a lipid bilayer on the outside of cells that provides the boundary between the interior and exterior of the cell (Jähnig, 1996). Directly underneath the membrane lies the cell cortex (also known as the actin cortex), which is made of a network of actin with associated myosin motors and binding proteins. The cortex plays an important role in determining cell shape. Biophysical properties of the cell boundary, such as tension and curvature, are typically composed of a contribution from the membrane and a contribution from the cortex. However, it is sometimes appropriate in modeling contexts to combine the two effects into one “proxy” effect that accounts for both the membrane and the cortex.

The cortical tension acts to change the surface area of the cell through myosin II motors pulling on the actin filaments within the cortex, generating stresses (Salbreux et al., 2012; Chugh et al., 2017). This force also acts to promote cell rounding in confined cells (Ramanathan et al., 2015). Recent studies have shown that cortical tension is affected by both myosin II levels and actin filament length (and, hence, cortical network thickness), with myosin contractility thought to account for most of the tension (Salbreux et al., 2012; Chugh et al., 2017; Ramanathan et al., 2015; Tinevez et al., 2009). Owing to the viscoelastic nature of the cortical contractile forces, they can lead to both tangential flows in the cortex or pressure generating normal forces (Salbreux et al., 2012; Ramanathan et al., 2015).

The simplest model of tension assumes the membrane and cortex act as a perfect elastic material that can be described by Hooke’s law. In terms of the cell area A and the cell’s resting/equilibrium area $A_{0}$, this suggests an energy of the form

$$E_{\mathrm{tension}}=k_{\mathrm{ten}}{\left(\frac{A-A_{0}}{A_{0}}\right)}^{2}\text{,}$$

where the $k_{\text{ten}}$ constant can be understood as a combination of the cortical and membrane stiffness (Le Guillou et al., 2009).

However, there are a number of more involved forms for the energy that may be more appropriate in certain models. First, the magnitude of the restorative force for a small deformation may be different depending on whether the membrane is compressed or extended. This can be modeled by an energy

$$E_{\text{tension}}=\left\{ \begin{array}{l} k_{\mathrm{ext}}{\left(\frac{A-A_{0}}{A_{0}}\right)}^{2}\;if\;A\geq A_{0}\\ k_{\mathrm{comp}}{\left(\frac{A-A_{0}}{A_{0}}\right)}^{2}\;if\;A<A_{0} \end{array}\right.\text{,}$$

where $k_{\mathrm{ext}}$ and $k_{\mathrm{comp}}$ are the extended and compressed stiffness constants, respectively.

Second, membranes can exhibit a degree of wrinkling or ruffling, which allows the membrane to be extended by small distances with little to no increase in the tension (Lecuit and Lenne, 2007). This also suggests there may be little tension when membranes are compressed (i.e., that $k_{\mathrm{comp}}$ is effectively zero). This would give yet another form of the energy:

$$E_{\text{tension}}=\left\{ \begin{array}{l} k_{\mathrm{ten}}{\left(\frac{A-A_{0}-A_{\text{spare}}}{A_{0}}\right)}^{2}\;if\;A\geq A_{0}+A_{\text{spare}}\\ 0\;\;\;\;\;\;\;\;\;\;\;otherwise \end{array}\right.\text{,}$$

where $A_{\text{spare}}$ is a measure of the “spare” membrane present within the membrane.

Finally, there are other factors that must sometimes be considered. These include the viscoelastic nature of the membrane (so that a purely elastic approximation may not be appropriate), the fact that the resting/equilibrium area $A_{0}$ need not be constant (but can change via, for example, exocytosis of internal membrane reserves), and the presence of tension fluctuations which can, for example, be included with Gaussian noise in the expression for tension (Kim et al., 2021).

### Membrane curvature

Defining the curvature of a 1D line is fairly simple: at any given point the curvature is the inverse of the radius of the osculating circle (which can be thought of as the circle that best fits the curve at that given point) (Farin and Sapidis, 1989). However, things are more interesting for a 2D surface such as a membrane. Then, for a given point p, there are an infinite number of curvatures, one for each line passing through p. From these curvatures it is useful to consider the maximum and minimum values, called the principal curvatures and typically denoted $\kappa_{1}$ and $\kappa_{2}$. By a famous 1760 theorem in differential geometry by Euler, the directions of the maximum and minimum curvatures are perpendicular (assuming $\kappa_{1}\neq \kappa_{2}$) and the curvature of any other direction is easily found from $\kappa_{1}$ and $\kappa_{2}$ (Deserno, 2007).

From the principal curvatures, two other curvatures can be defined: the mean curvature $H=\left(\kappa_{1}+\kappa_{2}\right)/2$ and the Gaussian curvature $K=\kappa_{1}\kappa_{2}$ (Deserno, 2007). Mean curvature, with dimensions of inverse length, is not an intrinsic measure of the surface curvature, but depends on how the surface is embedded in 3D space. This is in contrast to the Gaussian curvature, which has units of inverse squared length and is an intrinsic measure of surface curvature, independent of the embedding. A positive Gaussian curvature (where both principal curvatures have the same sign) corresponds to a (locally) spherical surface, whereas a negative Gaussian curvature (with opposite-signed principal curvatures) corresponds to a (locally) hyperbolic geometry (Bär, 2010).

The bending energy of a membrane is likely to be a complicated function of the precise surface shape. However, in the 1970s Helfrich derived a well-known approximate expression for the membrane energy in terms of the mean and Gaussian curvatures:

$$E_{\text{bending}}=\int \left[2k_{H}{\left(H-H_{0}\right)}^{2}+k_{G}K\right]dS\text{,}$$

where the integral is over the total membrane surface, $k_{H}$ is the bending modulus, and $k_{G}$ is the saddle splay modulus (Helfrich, 1973; Ni and Papoian, 2021). Here $H_{0}$ is the spontaneous membrane curvature, capturing the fact that the relaxed state for some membranes may not be flat.

Due to the Gauss-Bonnet theorem, the Gaussian curvature term $k_{G}\int KdS$ is a constant for a fixed membrane topology and so can often be neglected (Deserno, 2007). However, it is important to bear in mind that there are situations such as cell division when the topology changes, and it may be necessary to include this term (Ni and Papoian, 2021).

### Cell volume

The low compressibility of water means that the total cell volume is almost exactly conserved. Any attempt to alter the cell volume results in a pressure difference between the inside and outside of the cell and thus an overall restoring force, with a corresponding energy that is often modeled as

$$E_{\text{volume}}=k_{\text{vol}}{\left(\frac{V-V_{0}}{V_{0}}\right)}^{2}\text{,}$$

where V is the current cell volume, $V_{0}$ is the resting/equilibrium cell volume, and $k_{\text{vol}}$ is a compressibility constant (Honda et al., 2008). As with the tension force, more complicated functional forms are possible with, for example, different values of $k_{\text{vol}}$ depending on whether the cell is compressed ($V<V_{0}$) or extended ($V>V_{0}$).

Although this is a reasonable first approximation to $E_{\text{volume}}$, there are other important factors that must sometimes be considered, the most important being that (as with the membrane resting area $A_{0}$), the value of $V_{0}$ is not necessarily constant (for example during apoptosis or cleavages). Fluid is able to enter and leave the cell via a variety of mechanisms including diffusion, osmosis, aquaporins (specialized water channels), and (macro)pinocytosis (sometimes referred to as cell drinking) (Liu and Wintour, 2005; Ritter et al., 2021).

### Cell-cell adhesion

Interaction between cells is critical for self-organization of multicellular systems such as blastocysts. Throughout embryogenesis, cell-cell adhesion arises from, and is mediated by, a number of membrane-bound proteins. The force itself typically arises from specialized cell adhesion molecules such as epithelial cadherin (E-cadherin). Encoded by the CHD1 gene, E-cadherin is the adhesion molecule associated with filopodia behavior during compaction (Fierro-González et al., 2013; Samarage et al., 2015; Van Roy and Berx, 2008). The magnitude of cell-cell adhesion is often affected (and controlled) by other membrane-bound proteins such as Ephrin receptors (which bind to ephrin ligands and have been shown to contribute to differential adhesion). Different pairings from the Eph/Ephrin family trigger varying levels of adhesion, with some combinations such as EphB2/EphrinB2 triggering repulsion (Cang et al., 2021; Arvanitis and Davy, 2008).

The adhesion energy of a cell i in contact with a cell j can be modeled as

$$E_{\text{adhesion}}=-k_{\text{adh}}c_{\text{adh}}A_{ij}\text{,}$$

where $A_{ij}$ is the contact area between the two cells, $k_{\text{adh}}$ is the adhesion energy coefficient, and $c_{\text{adh}}$ is the adhesion molecule concentration (which can, for example, be dependent on Eph/Ephrin levels) (Krupinski et al., 2011; Le Guillou et al., 2009).

### The drag force

The sum of all the above effects (or some appropriate subset) leads to a total force, $F_{\text{total}}$. The resultant motion is then found by solving $F_{\text{total}}\;–\;F_{\text{drag}}=ma$, where $F_{\text{drag}}$ is the relevant drag force. Since biological systems at the length scale of single cells correspond to very low Reynolds number environments (typically $Re\;<\;10^{-4}$), it is usual to argue that inertial effects are negligible and to drop the ma term, leaving $F_{\text{drag}}=F_{\text{total}}$ (Fletcher et al., 2014).

The drag force can arise from a number of sources, including motion through the extracellular medium, the viscous nature of the cytoplasm, and interaction with neighboring cells. In low Reynolds number regimes, it is usual to assume that the drag force is proportional to velocity v (unlike at higher Reynolds numbers, where drag is typically proportional to $v^{2}$) (Krupinski et al., 2011). Furthermore, there is typically a dependence on the size of area being moved, although this is often neglected. Often it is easiest to simply assume $F_{\text{drag}}=\varepsilon v$, where ε is the effective mobility coefficient that can be found by fitting to existing data (Fletcher et al., 2014).

### Other forces

A number of other forces are sometimes considered, for example when considering the effect of the ZP or the blastocoel. One example is surface tension. This is not a new force but a combination of previously mentioned forces. After conflicting hypotheses of adhesion and cortical-tension-dependent surface tension, it is now thought to be a combination of the two (Foty and Steinberg, 2005; Manning et al., 2010). Surface tension of a cell has a large impact on its resulting shape, and its modulation can result in important morphological processes such as compaction and internalization, with more cohesive cells enveloped by the cells with lower adhesion (Samarage et al., 2015; Lecuit and Lenne, 2007; Goldstein and Kiehart, 2015).

Another example is the constraint imposed by the ZP, whose shape is believed to influence packing of blastomeres. This constraint has also been linked to positioning of the blastocoel and the ICM on opposite sides of the blastocyst, forming the embryonic axis (Honda et al., 2008; Kurotaki et al., 2007). The simplest implementations of the ZP simply impose an energy step at the ZP boundary (Honda et al., 2008; Giammona and Campàs, 2021).

Other processes, such as the role of fluctuations, have also been considered in recent years (Yanagida et al., 2022; Kim et al., 2021). For example, Kim et al. have included tension fluctuations when modeling embryonic tissues as active foams, showing that such fluctuations could be responsible for stress relaxation in tissues helping to control tissue fluidization.

The final effect we mention is that considering the fluid dynamics of blastocoel formation. Dumortier et al. (2019) have developed a model of blastocoel formation that is based on hydraulic fracturing of cell-to-cell contacts and coarsening of the subsequent microlumens. It is argued that the flow Q of fluid between microlumens is controlled by the pressure gradient ΔP, with $Q=-d^{3}\Delta P/12\eta$, where d is the scale of the intercellular space and η the viscosity. The pressure gradient itself is given by Laplace’s law, $P-P_{\mathrm{ref}}=2\gamma /R$, with R being the curvature radius of the microlumen, γ the tension, and $P_{\mathrm{ref}}$ some reference pressure.

## Existing models of blastocyst formation

We now discuss previously published models of embryogenesis, focusing on the theoretical frameworks used, the forces and biophysical details considered, the model outputs, and the results and predictions. Since the modeling framework can have a profound influence on the output, we also directly compare the different models, highlighting their various advantages and disadvantages. Although some models span multiple stages of blastocyst formation, we choose to organize our discussion into five stages, with some models covered in multiple sections.

### The fertilized egg to the morula

The arrangement of early blastomeres has been the subject of several biophysical models. Krupinski et al. modeled blastomeres based on a model by Dallon and Othmer, which uses a similar framework to the particle-potential models discussed above (Krupinski et al., 2011; Dallon and Othmer, 2004). The cells are confined by an ellipsoidal ZP and are subjected to three forces: an elastic interaction between cells, adhesion, and drag. Cell types, and hence cell properties, are informed by a genetic network, with both symmetric and asymmetric division considered. They found that even less than a 1% difference in the axis lengths of the ZP was enough to position the blastomeres along the ZP's long axis. Another model by Le Guillou et al. is based on a 3D cellular Potts model and is also able to recapitulate blastocyst development up to the 16-cell stage (Le Guillou et al., 2009). Unlike the Krupinski et al. model, this model uses a spherical ZP and is able to produce simulated development that is robust to small changes in model parameters. Le Guillou et al. compared their simulations with confocal images of mouse embryos, focusing on embryo shape and cell arrangement, and found qualitative agreement up to the 16-cell stage. It is worth pointing out that this agreement is not perfect, and the authors note that further calibration and validation are needed before their model is able to make meaningful predictions.

Giammona and Campàs (2021) focused specifically on the effect of the physical confinement imposed by the ZP as well as the impact of division rules on the final geometry of the four-cell mammalian embryo. Cells are modeled using a similar approach to the particle-potential framework and their shape is defined using a 3D Voronoi diagram, with the governing equations solved via the Euler-Maruyama method (Burrage et al., 2000). In the non-confined cases (without a ZP), the resulting orientation of blastomeres was found to depend on time between divisions, which becomes a measure of whether the cells have time to reach dynamic equilibrium. However, in the confined case, the model suggests it is the shape of the ZP shell that determines the equilibrium packing formation, negating the effect of division rules as well as increasing the rate of the packing process. This model implied that it is the shell that is responsible for eliminating packing degeneracy, thereby ensuring that blastomeres have the correct orientation.

The existing work on modeling up to the 16-cell stage highlights the importance of the exact shape and properties of the ZP. Although for some mammals a ZP is not necessary for blastocyst formation or implantation, the ZP does seem to be related, at least in part, to the different packing geometries observed in different species (Krupinski et al., 2011; Honda et al., 2008; Kurotaki et al., 2007; Rottmann and Lampeter, 1981). Future modeling will need to carefully examine how best to represent and implement the ZP. It has also been suggested that the mechanical properties of these early blastomeres can be the predictors of embryo viability, which may be an additional avenue for investigation of models in the future (Yanez et al., 2016).

### Compaction and internalization

Modeling of the driving forces behind compaction and internalization have been ongoing for decades. Goel et al. (1986) proposed that, in mammals, both processes are driven by minimization of the surface energy of the embryo. This model is based on energy minimization using a vertex-type model. Shared vertices along cell membranes move if energetically favorable, leading to overall energy minimization of the system. The simulations were able to mimic both compaction and internalization using surface energy, which suggests that the biological process may be driven by a similar mechanism. In the aforementioned cellular Potts model by Le Guillou et al. (2009), compaction is modeled by altering the adhesion coefficient in the Hamiltonian which, given the hypothesis that surface tension is related to adhesion, may also be interpreted as altering the surface tension (Manning et al., 2010).

Maître et al. numerically simulated the mammalian blastocyst in 3D using a multimaterial mesh-based surface-tracking method (Maître et al., 2016; Da et al., 2014; Firmin et al., 2022; Turlier and Maître, 2015). They hypothesized that compaction and internalization were due to differing contractility between cells, which they implemented using surface tension. They showed that cells will internalize given a certain level of tension asymmetry between cells for a given compaction parameter α, which they define as $\gamma_{c}/2\gamma_{2}$, where $\gamma_{c}$ is the surface tension between cells and $\gamma_{2}$ is the surface tension between a particular cell and the extracellular matrix. They then tested the model predictions by tracking blastomere positions and measuring their surface tension with microaspiration. Based on the model and subsequent experiments, they suggest that differences in cell contractility between polar and apolar cells is enough to drive the internalization process.

Future work that models compaction and/or internalization will need to incorporate cell motion and will most likely need to consider differing surface tension, possibly through a combination of adhesion and tensile properties. This will also need to include multiple cell types, at least trophectoderm and the ICM. An extension of this may be to include differing surface properties throughout the membrane of individual cells in order to account for the apical domain and cell-cell contacts.

### Trophectoderm differentiation

The elastic sphere representation of blastomeres in Krupinski et al. (2011) was also used to study the mechanisms behind trophectoderm differentiation. At the time of this work it was not known whether trophectoderm cell fate was decided by cell position or cell polarity. Krupinski et al. model these two scenarios using a simplified gene network involving Oct4 and Cdx2. Outer cells (determined by a Voronoi diagram) experience an increase in Cdx2 levels, which downregulates their Oct4 levels. This model was found to be robust and to agree with experimental findings of higher Cdx2 levels in outer cells (Ralston and Rossant, 2008). However, the polarity-based model was found to be less robust and had no mechanism for dealing with incorrectly placed high-Cdx2-expressing cells. The position-based approach is now the most common method for modeling trophectoderm differentiation (Herrera-Delgado and Maître, 2021).

Nissen et al. (2017) simulated blastocyst formation through a phenomenological agent-based method by modeling cells as off-lattice cells in two dimensions that interact through an energy potential. The outer cells, which because of increased size are modeled as two circles, are defined by nearest-neighbor counting and assigned a polarity that points radially outward from the center of the blastocyst. The attraction factor included in the governing potential means that cell interactions are polarity dependent in order to recreate the tight junctions formed at the trophectoderm junctions. Without this polarity rule, cells did not form a shell-like layer and instead clustered together, which agreed with experimental findings in mouse mutants with polarity defects.

Because the differentiation process is controlled by GRNs, purely biophysical models (without any GRN component) will often struggle when probing this process. Therefore, models that probe lineage specification will most typically need to include GRNs alongside biophysical considerations.

### Blastocoel formation

Honda et al. (2008) used a 3D vertex model to simulate positioning of the blastocoel and the subsequent creation of the embryonic-abembryonic (Em-Ab) axis. The model does not differentiate between cell types, only between cells and the blastocoel (which is assumed to be lined by a membrane that is firmer than that of the cells). It relies on the geometry of the ZP to position the blastocoel between the endoderm and trophectoderm cells. An ellipsoidal ZP was able to orient the ICM on the long axis of the shell, whereas a spherical ZP left the axis of the embryo unfixed. Interestingly, even without distinct properties for the ICM and trophectoderm, the model can reproduce the ratio of the number of inner to outer cells as well as the fact that trophectoderm cells are significantly larger than those of the ICM. Honda et al. were able to conclude that the mechanical constraint of the ZP was sufficient to produce the Em-Ab axis, supporting other work that suggests a lack of pre-patterning prior to the blastocyst stage (Alarcón and Marikawa, 2003; Kurotaki et al., 2007; Motosugi et al., 2005). The authors note that future models would benefit from including additional factors, particularly cell division, microcavity formation, and different cell properties for distinct cell types.

Krupinski et al. (2011) also modeled blastocoel positioning. Their simulation starts at the 32-cell stage after the trophectoderm and ICM cell lineage has already differentiated. they then implement a slowly expanding spherical region inside the ICM as the blastocoel. They modulate the adhesion forces between cells so that ICM cells preferentially adhere to themselves but have reduced adhesion to trophectoderm cells. As in the Honda et al. model, they were able to achieve alignment of the Em-Ab axis with the long axis of the embryo by using a slightly ellipsoidal ZP (Honda et al., 2008).

Dumortier et al. (2019) described blastocoel formation in significantly finer detail by including the network of microlumens (which they observed in mouse embryos using high-resolution imaging) that are proposed to occur through hydraulic fracturing of cell-to-cell contacts. By using the Young-Laplace equation, they related the pressure of the lumens to the contractility and curvature of different cell types. Unlike previous models, positioning of the blastocoel between the trophectoderm and ICM was not dependent on an ellipsoidal ZP but instead arose simply from the properties ascribed to each cell type. The increased contractility of cells in the ICM caused an increase in lumen pressure compared with those formed at the trophectoderm cell border. This model was also able to capture the swelling phase in the microlumens before they discharged into other lumens. The Myh9 protein, which affects surface tensions, was knocked out maternally in chimeras to investigate how blastocoel positioning was affected. This showed that differences in Myh9 levels were sufficient for blastocoel positioning, agreeing with their simulations.

It is still unclear to what extent a non-spherical ZP is important for the positioning of the blastocoel, since only some models require this. It would be interesting to build a model that includes both an ellipsoidal ZP and distinct contractility properties for different cell types in order to compare their respective impact on blastocoel position as well as explore the possible impact of the chosen modeling framework.

### Inner cell mass differentiation and sorting

Krupinski et al. (2011) also simulated cell sorting of the epiblast and primitive endoderm (hypoblast). They used differential adhesion with either a static blastocoel (which creates a force on Gata6-expressing cells) or a dynamic blastocoel (which exerts a pressure on the neighboring cells). A random component to cell motion was found to increase the efficiency of cell sorting. In the static blastocoel model the Gata6 force was found to be critical for cell sorting, whereas in the dynamic case both the pressure force and preferential adhesion between epiblast and trophectoderm cells was necessary for correct blastocoel placement. No apoptosis was included, but its inclusion may have had a large impact on misplaced cells.

Cang et al. (2021) used single-cell gene expression data to inform their model of trophectoderm, epiblast, and primitive endoderm differentiation dynamics. Cells were constrained in a spherical geometry, and the model was implemented with the subcellular element method. Their findings concentrated on elucidating details of the GRNs that govern the epiblast/primitive endoderm (Epi/PrE) specification process. According to their model, Eph4/EphrinB2 is likely a driving mechanism underpinning selective adhesion, and attenuation of Fgf signaling is required after sorting of the ICM.

In the off-lattice model of Nissen et al. (2017), there are three more rules that work together to reproduce the Epi/PrE specification process. The first is that FGF regulates the salt-and-pepper pattern (implemented by switching cell fate when there are too many similar neighbors), the second is related to differential adhesion (by altering the attraction factor), and the third concerns apoptosis during primitive endoderm specification (cells are removed when out of place). Interestingly, just these three simple phenomenological rules along with the aforementioned polarization rule lead to a 79% success rate in blastocyst development. The model was able to reproduce the correct ratio of cell lineages, the rate of apoptosis in the ICM, and the outcome of scaling experiments. Low FGF levels in the model lead to an ICM composed only of epiblast cells, agreeing with the results from real FGF-inactivated embryos (Krawchuk et al., 2013; Cheng et al., 1998; Kang et al., 2013; Wilder et al., 1997). ICMs consisting of only primitive endoderm cells formed from a constant on-state of FGF, which matches the results of experiments that used high levels of FGF (Yamanaka et al., 2010; Krawchuk et al., 2013; Schrode et al., 2014). With no differential adhesion, the blastocyst failure rate in the model is close to 100%, again agreeing with results showing that deletion of adhesion molecules leads to ICM sorting failure (Stephens et al., 1995; Gao et al., 2004; Morris et al., 2002; Smyth et al., 1999; Yang et al., 2002). Finally, without apoptosis there are substantially more misplaced cells.

Yanagida et al. used a 3D model in which cells are treated as a collection of elements that interact through nearest-neighbor Morse potentials, with the addition of subcellular mechanics as well as tissue-scale considerations. They first showed that experimentally measured levels of cell-cell affinity (a combination of adhesion, cell surface tension, and interfacial tension) could not by themselves explain ICM sorting. Subsequently, surface fluctuations were implemented in the model, with the ratio of fluctuations between epiblast and primitive endoderm cells determined experimentally. This led to robust sorting. As an experimental validation, they then created cell aggregates with a range of surface fluctuations, finding that the final cell position was indeed highly dependent on the size of the fluctuations (Revell et al., 2019; Yanagida et al., 2022).

Both Krupinski et al. and Yanagida et al. suggest that differential adhesion between cells is not enough by itself to explain sorting of the ICM (Krupinski et al., 2011; Yanagida et al., 2022). Whether this can be rectified by including processes such as directional blastocoel forces and apoptosis is still not clear (Plusa et al., 2008). Yanagida et al. instead suggest that non-equilibrium characteristics (active forces) are crucial to the understanding of morphogenic processes. Active forces have not been widely considered in models of blastocyst development and will be a fascinating avenue for future research (Yanagida et al., 2022; Revell et al., 2019).

### Model validation

For modeling to be useful, it is essential that it is informed by and fitted to biological data. Model validation, meaning testing that a model accurately represents reality, is also crucial (Henninger et al., 2010). Of course, models can never truly be validated, only invalidated: it is always possible that a given model replicates the process of interest while still failing to capture accurately the true biophysics and biology at play (Anderson and Papachristodoulou, 2009).

Model predictions should always, if possible, be compared with real biological data. Importantly, predictions that are invalidated are not necessarily unhelpful. In fact, they are often significantly more useful than validated predictions because they can give further insight into a system by, for example, indicating that a key process is missing from the model.

Models that focus on the physical shape of cells can often be directly tested by using microscopy of real blastocysts. This typically involves some level of automatic image analysis to quantify important properties such as cell size, cell shape, and membrane curvature. Mutant cells with altered morphological characteristics can also be useful tests as, for example, in the cell aggregates with varying surface fluctuations used in Yanagida et al. (2022) and the Myh9 knockout chimeras employed by Dumortier et al. (2019).

Cell forces can sometimes also be directly measured *in vitro* as an additional means of validation. For example, cell microaspiration was used by Maître et al. to measure cell surface tensions of internalizing cells, confirming that the tension asymmetries between inner and outer cells were above the threshold for internalization predicted by their model (Maître et al., 2016).

Given the limitations on human embryo research, blastocyst model validation up until now has usually been carried out on animal models, typically mouse embryos. The recent creation of human blastoid models offers the opportunity to change this by providing more human-focused data. Blastoids are not constrained by the same ethical limitations that apply to human embryos (Human Fertilisation and Embryology Act, 1990 and 2008), making it substantially easier to obtain high-quality image data via a range of techniques (Lovell-Badge et al., 2021). Large numbers of genetically identical datasets can also be provided by blastoids, something that is typically impossible with human embryos. This makes it easier to probe the relative importance of environmental noise versus genetic mutation and aids the gathering of sufficient data to validate model predictions (Daoud et al., 2021). Reduced ethical restrictions also make it possible to create genetically altered blastoids so that, for example, cell lineage can easily be identified (Yanagida et al., 2021; Mehta, 2014).

## Discussion and the future

In this review, we have discussed the biology, biophysics, and modeling frameworks of pre-implantation development, stressing the importance and utility of multidisciplinary approaches in this area. We reviewed existing models of early embryogenesis, covering all stages from the single fertilized egg to the mature blastocyst, including both models of individual events and models that span multiple stages. In each case we have tried to explain the advantages and disadvantages of the chosen modeling framework while focusing on the biophysical understanding and predictions that emerged from each model.

Multidisciplinary approaches that study development by supplementing traditional biological approaches with physics, chemistry, mathematics, engineering, and computing are becoming increasingly common. Bringing together researchers with diverse backgrounds, training, and skill sets can generate ideas and approaches that are not possible by isolated groups (Cech and Rubin, 2004; Gardy and Brinkman, 2003). For example, cross-disciplinary approaches, particularly those that include predictive modeling, can lead to novel understanding, often at a fundamental level, that can be difficult to attain otherwise. Other advantages include quicker, cheaper, more humane research that uses fewer animals. Furthermore, it is often possible to test hypotheses and make progress that is simply not possible with traditional experiments, including finding connections between diverse organisms, discovering underlying rules, and studying extreme situations (for example with unphysical parameter values).

There are several important factors to consider when designing a biophysical or mathematical model. Arguably the most important is the choice of model framework. This can be guided by the types of questions that are being addressed, the desired outputs, and the types of predictions required. For example, if the focus is cell sorting, it is important to consider a framework (such as cellular Potts or vertex models) that will allow for cells to be rearranged and to interact with their neighbors. When the framework is fixed, it is then necessary to decide which biophysical and biological ingredients to include. Models that attempt to include every component and process are rarely useful, not least because they tend to lack predictive power. Instead only the most relevant factors should be included, while bearing in mind the need to capture the real biological system as accurately as possible. For example, a study of the biophysics of blastocoel formation needs to consider fluid dynamics and the force that the developing blastocoel exerts on neighboring cells, but may not need to explicitly model detailed GRNs.

As demonstrated by the recent increase in the number of models of embryogenesis, there is substantial scope for future modeling in this area. There are a variety of forms these models could take. First, novel biological cellular models and improved experimental methods may well mean that new biophysical modeling can be driven by data in a way that previously has not been possible. It is likely that the available data will soon reach unprecedented levels so that additional theoretical options such as machine learning will become applicable. Second, future research may focus on understanding multiple stages of development within single models. For example, there is an opportunity for extending existing cellular Potts models of the morula to cover blastocyst development (see Figure 3). It is important to remember that including multiple stages within one model is not always advantageous, although it will allow underlying principles that operate throughout the whole of embryogenesis to be investigated. Third, while some work has already started to do this, there is further scope for adding increased realism to some models, such as by including stochastic effects or moving from two to three dimensions. Finally, based on existing initial work in this direction, future models may benefit from explicitly including the role of GRNs, which are an integral part of cell specification (Cang et al., 2021; Krupinski et al., 2011). One advantage of this is the ability to then simulate dysregulated GRNs, which would allow possible mechanisms behind blastocyst failure to be explored (Karlebach and Shamir, 2008).

We have exclusively covered the earliest stages of embryogenesis up to the mature blastocyst, but one of the chief advantages of modeling is that similar frameworks and techniques are equally applicable to later stages. Study of post-implantation development of human embryos is necessarily heavily restricted by ethical and technical factors (such as the 14-day rule). The developmental period of 7–28 days is known as the “black box” of human development. This has meant that mouse models have been heavily relied upon for post-implantation research, but this has resulted in only limited conclusions for humans due to differences in genetics and structure (Rossant, 2015). Recent biological model systems, such as embryoids, have begun to be used to study later-stage embryos (Zheng et al., 2021) and, in combination with the types of biophysical models we have discussed here, are likely to aid fundamental understanding of the processes involved in later-stage embryo development in the near future.

A significant drawback in model development has traditionally been the difficulty in obtaining high-quality, high-resolution data to inform, drive, and fit models. Mouse models can only go so far, since caution must be taken when comparing with other organisms such as humans. For example, while both mouse and human blastocysts contain trophectoderm, epiblast, and primitive endoderm cells, there are important differences in the timing and lineage specification processes (Rossant, 2015). Blastoids offer a valuable experimental model to study early human embryo development. They make it possible to collect significant amounts of data on key parameters such as the magnitude of intercellular forces and cell surface tensions, and thus inform and test biophysical models without relying on human embryos as the primary research system. Blastoids provide a unique opportunity to combine cell-based models with high-throughput data, allowing improved parameter fitting and the possibility of using of machine-learning techniques.

Modeling in biology (including biophysical and mathematical modeling), especially when used in a multidisciplinary context, has become a hallmark of modern research. The study of the earliest stages of embryogenesis, from the single fertilized egg to the blastocyst, is no exception. Future advances in fundamental biological knowledge, assisted human conception such as IVF, and global food security will require the types of focused, data-driven modeling of the embryo that we have discussed here, as we seek a deeper understanding of development throughout biology.

## Author contributions

L.W. created the cellular Potts model. A.D. provided the blastoid bright-field image. A.C., K.T.-A., and D.M.R. wrote the paper. A.C., G.G., A.S., K.T.-A., and D.M.R. edited and reviewed the paper.

## References

[bib1] Achache H., Revel A. (2006). Endometrial receptivity markers, the journey to successful embryo implantation. Hum. Reprod. Update.

[bib2] Ai B.-q., Xu G.-h., Li J.-j., He Y.-f. (2022). Rectification of self-propelled cells in confluent tissues. Europhys. Lett..

[bib3] Alarcón V.B., Marikawa Y. (2003). Deviation of the blastocyst axis from the first cleavage plane does not affect the quality of mouse postimplantation development. Biol. Reprod..

[bib4] Allena R., Mouronval A.-S., Aubry D. (2010). Simulation of multiple morphogenetic movements in the drosophila embryo by a single 3d finite element model. J. Mech. Behav. Biomed. Mater..

[bib5] Alt S., Ganguly P., Salbreux G. (2017). Vertex models: from cell mechanics to tissue morphogenesis. Philos. Trans. R. Soc. Lond. B Biol. Sci..

[bib6] Anderson J., Papachristodoulou A. (2009). On validation and invalidation of biological models. BMC Bioinf..

[bib7] Arroyo M., Trepat X. (2019). Embryonic self-fracking. Science.

[bib8] Arvanitis D., Davy A. (2008). Eph/ephrin signaling: networks. Genes Dev..

[bib9] Bär C. (2010).

[bib10] Barton D.L., Henkes S., Weijer C.J., Sknepnek R. (2017). Active vertex model for cell-resolution description of epithelial tissue mechanics. PLoS Comput. Biol..

[bib11] Beddington R.S., Robertson E.J. (1999). Axis development and early asymmetry in mammals. Cell.

[bib12] Bessonnard S., De Mot L., Gonze D., Barriol M., Dennis C., Goldbeter A., Dupont G., Chazaud C. (2014). Gata6, nanog and erk signaling control cell fate in the inner cell mass through a tristable regulatory network. Development.

[bib13] Bi D., Lopez J.H., Schwarz J.M., Manning M.L. (2015). A density-independent rigidity transition in biological tissues. Nat. Phys..

[bib14] Bi D., Yang X., Marchetti M.C., Manning M.L. (2016). Motility-driven glass and jamming transitions in biological tissues. Phys. Rev. X.

[bib15] Bidhendi A.J., Geitmann A. (2018). Finite element modeling of shape changes in plant cells. Plant Physiol..

[bib16] Boromand A., Signoriello A., Ye F., O’Hern C.S., Shattuck M.D. (2018). Jamming of deformable polygons. Phys. Rev. Lett..

[bib17] Boroviak T., Stirparo G.G., Dietmann S., Hernando-Herraez I., Mohammed H., Reik W., Smith A., Sasaki E., Nichols J., Bertone P. (2018). Single cell transcriptome analysis of human, marmoset and mouse embryos reveals common and divergent features of preimplantation development. Development.

[bib18] Wayne Brodland G., Chen H.H. (2000). The mechanics of cell sorting and envelopment. J. Biomech..

[bib19] Brunt L.H., Norton J.L., Bright J.A., Rayfield E.J., Hammond C.L. (2015). Finite element modelling predicts changes in joint shape and cell behaviour due to loss of muscle strain in jaw development. J. Biomech..

[bib20] Burrage K., Burrage P., Mitsui T. (2000). Numerical solutions of stochastic differential equations–implementation and stability issues. Journal of Computational and Applied Mathematics.

[bib21] Burton G. (1992). The First Twelve Weeks of Gestation.

[bib22] Camley B.A., Rappel W.-J. (2017). Physical models of collective cell motility: from cell to tissue. J. Phys. D Appl. Phys..

[bib23] Cang Z., Wang Y., Wang Q., Cho K.W.Y., Holmes W., Nie Q. (2021). A multiscale model via single-cell transcriptomics reveals robust patterning mechanisms during early mammalian embryo development. PLoS Comput. Biol..

[bib24] Cech T.R., Rubin G.M. (2004). Nurturing interdisciplinary research. Nat. Struct. Mol. Biol..

[bib25] Chaturvedi R., Huang C., Kazmierczak B., Schneider T., Izaguirre J.A., Glimm T., Hentschel H.G.E., Glazier J.A., Newman S.A., Alber M.S. (2005). On multiscale approaches to three-dimensional modelling of morphogenesis. J. R. Soc. Interface.

[bib26] Chazaud C., Yamanaka Y. (2016). Lineage specification in the mouse preimplantation embryo. Development.

[bib27] Chen X., Brodland G.W. (2008). Multi-scale finite element modeling allows the mechanics of amphibian neurulation to be elucidated. Phys. Biol..

[bib28] Cheng A.M., Saxton T.M., Sakai R., Kulkarni S., Mbamalu G., Vogel W., Tortorice C.G., Cardiff R.D., Cross J.C., Muller W.J., Pawson T. (1998). Mammalian grb2 regulates multiple steps in embryonic development and malignant transformation. Cell.

[bib29] Chugh P., Clark A.G., Smith M.B., Cassani D.A.D., Dierkes K., Ragab A., Roux P.P., Charras G., Salbreux G., Paluch E.K. (2017). Actin cortex architecture regulates cell surface tension. Nat. Cell Biol..

[bib30] Cimadomo D., Sosa Fernandez L., Soscia D., Fabozzi G., Benini F., Cesana A., Dal Canto M.B., Maggiulli R., Muzzì S., Scarica C. (2022). Inter-centre reliability in embryo grading across several ivf clinics is limited: implications for embryo selection. Reprod. Biomed. Online.

[bib31] Cockburn K., Rossant J. (2010). Making the blastocyst: lessons from the mouse. J. Clin. Invest..

[bib32] Conte V., Muñoz J.J., Miodownik M. (2008). A 3d finite element model of ventral furrow invagination in the drosophila melanogaster embryo. J. Mech. Behav. Biomed. Mater..

[bib33] Da F., Batty C., Grinspun E. (2014). Multimaterial mesh-based surface tracking. ACM Trans. Graph..

[bib34] Dahl-Jensen S., Grapin-Botton A. (2017). The physics of organoids: a biophysical approach to understanding organogenesis. Development.

[bib35] Dallon J.C., Othmer H.G. (2004). How cellular movement determines the collective force generated by the dictyostelium discoideum slug. J. Theor. Biol..

[bib36] Pereira Daoud A.M., Dondorp W.J., de Wert G.M.W.R. (2021). The closer the knit, the tighter the fit: conceptual and ethical issues of human embryo modelling. Reprod. Biomed. Online.

[bib37] Davidson L.A., Koehl M.A., Keller R., Oster G.F. (1995). How do sea urchins invaginate? using biomechanics to distinguish between mechanisms of primary invagination. Development.

[bib38] Deserno M. (2007). Fluid Lipid Membranes–A Primer. http://www.cmu.edu/biolphys/deserno/pdf/membrane%20theory.%20pdf.

[bib39] Drasdo D., Hoehme S., Block M. (2007). On the role of physics in the growth and pattern formation of multi-cellular systems: what can we learn from individual-cell based models?. J. Stat. Phys..

[bib40] Drasdo D., Höhme S. (2005). A single-cell-based model of tumor growth in vitro: monolayers and spheroids. Phys. Biol..

[bib41] Dumortier J.G., Le Verge-Serandour M., Tortorelli A.F., Mielke A., De Plater L., Turlier H., Maître J.L. (2019). Hydraulic fracturing and active coarsening position the lumen of the mouse blastocyst. Science.

[bib42] Durand M. (2021). Large-scale simulations of biological cell sorting driven by differential adhesion follow diffusion-limited domain coalescence regime. PLoS Comput. Biol..

[bib43] Eckert J.J., Fleming T.P. (2008). Tight junction biogenesis during early development. Biochim. Biophys. Acta.

[bib44] Farin G., Sapidis N. (1989). Curvature and the fairness of curves and surfaces. IEEE Comput. Graph. Appl..

[bib45] Fierro-González J.C., White M.D., Silva J.C., Plachta N. (2013). Cadherin-dependent filopodia control preimplantation embryo compaction. Nat. Cell Biol..

[bib46] Firmin J., Ecker N., Danon D.R., Lange V.B., Turlier H., Patrat C., Maître J.-L. (2022). Mechanics of human embryo compaction. bioRxiv.

[bib47] Firmin J., Maître J.L. (2021). Morphogenesis of the human preimplantation embryo: bringing mechanics to the clinics. Semin. Cell Dev. Biol..

[bib48] Fletcher A.G., Osterfield M., Baker R.E., Shvartsman S.Y. (2014). Vertex models of epithelial morphogenesis. Biophys. J..

[bib49] Foty R.A., Steinberg M.S. (2005). The differential adhesion hypothesis: a direct evaluation. Dev. Biol..

[bib50] Frankenberg S.R., de Barros F.R.O., Rossant J., Renfree M.B. (2016). The mammalian blastocyst. Wiley Interdiscip. Rev. Dev. Biol..

[bib51] Gao F., Shi H.Y., Daughty C., Cella N., Zhang M. (2004). Maspin plays an essential role in early embryonic development. Development.

[bib52] Gardy J., Brinkman F. (2003). The benefits of interdisciplinary research: our experience with pathogen bioinformatics. Science Next Wave.

[bib53] Gerri C., Menchero S., Mahadevaiah S.K., Turner J.M.A., Niakan K.K. (2020). Human embryogenesis: a comparative perspective. Annu. Rev. Cell Dev. Biol..

[bib54] Giammona J., Campàs O. (2021). Physical constraints on early blastomere packings. PLoS Comput. Biol..

[bib55] Gillies T.E., Cabernard C. (2011). Cell division orientation in animals. Curr. Biol..

[bib56] Glen C.M., Kemp M.L., Voit E.O. (2019). Agent-based modeling of morphogenetic systems: advantages and challenges. PLoS Comput. Biol..

[bib57] Goel N.S., Doggenweiler C.F., Thompson R.L. (1986). Simulation of cellular compaction and internalization in mammalian embryo development as driven by minimization of surface energy. Bull. Math. Biol..

[bib58] Goldstein B., Kiehart D.P. (2015). Moving inward: establishing the mammalian inner cell mass. Dev. Cell.

[bib59] Graner F., Glazier J.A. (1992). Simulation of biological cell sorting using a two-dimensional extended Potts model. Phys. Rev. Lett..

[bib60] Hasty J., McMillen D., Isaacs F., Collins J.J. (2001). Computational studies of gene regulatory networks: in numero molecular biology. Nat. Rev. Genet..

[bib61] Heisenberg C.-P., Bellaïche Y. (2013). Forces in tissue morphogenesis and patterning. Cell.

[bib62] Helfrich W. (1973). Elastic properties of lipid bilayers: theory and possible experiments. Z. Naturforsch. C..

[bib63] Henninger H.B., Reese S.P., Anderson A.E., Weiss J.A. (2010). Validation of computational models in biomechanics. Proc. Inst. Mech. Eng. H.

[bib64] Hernandez Gifford J.A., Gifford C.A. (2013). Role of reproductive biotechnologies in enhancing food security and sustainability. Animal Frontiers.

[bib65] Herrera-Delgado E., Maître J.L. (2021). A theoretical understanding of mammalian preimplantation development. Cells Dev..

[bib66] Hervas-Raluy S., Garcia-Aznar J.M., Gomez-Benito M.J. (2019). Modelling actin polymerization: the effect on confined cell migration. Biomech. Model. Mechanobiol..

[bib67] Honda H., Motosugi N., Nagai T., Tanemura M., Hiiragi T. (2008). Computer simulation of emerging asymmetry in the mouse blastocyst. Development.

[bib68] (1990). Human Fertilisation and Embryology Act 1990.

[bib69] (2008). Human Fertilisation and Embryology Act 2008.

[bib70] Iwata K., Yumoto K., Sugishima M., Mizoguchi C., Kai Y., Iba Y., Mio Y. (2014). Analysis of compaction initiation in human embryos by using time-lapse cinematography. J. Assist. Reprod. Genet..

[bib71] Jähnig F. (1996). What is the surface tension of a lipid bilayer membrane?. Biophys. J..

[bib72] Jamali Y., Azimi M., Mofrad M.R.K. (2010). A sub-cellular viscoelastic model for cell population mechanics. PLoS One.

[bib73] Johnson M.H., Ziomek C.A. (1981). The foundation of two distinct cell lineages within the mouse morula. Cell.

[bib74] Kagawa H., Javali A., Khoei H.H., Sommer T.M., Sestini G., Novatchkova M., Scholte op Reimer Y., Castel G., Bruneau A., Maenhoudt N. (2022). Human blastoids model blastocyst development and implantation. Nature.

[bib75] Kang M., Piliszek A., Artus J., Hadjantonakis A.-K. (2013). Fgf4 is required for lineage restriction and salt-and-pepper distribution of primitive endoderm factors but not their initial expression in the mouse. Development.

[bib76] Karlebach G., Shamir R. (2008). Modelling and analysis of gene regulatory networks. Nat. Rev. Mol. Cell Biol..

[bib77] Kida N., Morishita Y. (2018). Continuum mechanical modeling of developing epithelial tissues with anisotropic surface growth. Finite Elem. Anal. Des..

[bib78] Kim S., Pochitaloff M., Stooke-Vaughan G.A., Campàs O. (2021). Embryonic tissues as active foams. Nat. Phys..

[bib79] Krawchuk D., Honma-Yamanaka N., Anani S., Yamanaka Y. (2013). Fgf4 is a limiting factor controlling the proportions of primitive endoderm and epiblast in the icm of the mouse blastocyst. Dev. Biol..

[bib80] Krupinski P., Chickarmane V., Peterson C. (2011). Simulating the mammalian blastocyst-molecular and mechanical interactions pattern the embryo. PLoS Comput. Biol..

[bib81] Krupinski P., Chickarmane V., Peterson C. (2012). Computational multiscale modeling of embryo development. Curr. Opin. Genet. Dev..

[bib82] Kuijk E.W., van Tol L.T.A., Van de Velde H., Wubbolts R., Welling M., Geijsen N., Roelen B.A.J. (2012). The roles of fgf and map kinase signaling in the segregation of the epiblast and hypoblast cell lineages in bovine and human embryos. Development.

[bib83] Kurotaki Y., Hatta K., Nakao K., Nabeshima Y.-i., Fujimori T. (2007). Blastocyst axis is specified independently of early cell lineage but aligns with the zp shape. Science.

[bib84] Le Guillou L., Dard N., Glisse J., Maro B., Louvet-Vallée S., Laforge B. (2009). A 3d mechanical model of the early mammalian embryo. J. Biol. Phys. Chem..

[bib85] Le Verge-Serandour M., Turlier H. (2022). Blastocoel morphogenesis: a biophysics perspective. Semin. Cell Dev. Biol..

[bib86] Lecuit T., Lenne P.-F. (2007). Cell surface mechanics and the control of cell shape, tissue patterns and morphogenesis. Nat. Rev. Mol. Cell Biol..

[bib87] Lim H.Y.G., Plachta N. (2021). Cytoskeletal control of early mammalian development. Nat. Rev. Mol. Cell Biol..

[bib88] Liu A., Rugonyi S., Pentecost J.O., Thornburg K.L. (2007). Finite element modeling of blood flow-induced mechanical forces in the outflow tract of chick embryonic hearts. Comput. Struct..

[bib89] Liu H., Wintour E.M. (2005). Aquaporins in development–a review. Reprod. Biol. Endocrinol..

[bib90] Liu X., Tan J.P., Schröder J., Aberkane A., Ouyang J.F., Mohenska M., Lim S.M., Sun Y.B.Y., Chen J., Sun G. (2021). Modelling human blastocysts by reprogramming fibroblasts into iblastoids. Nature.

[bib91] Lovell-Badge R., Anthony E., Barker R.A., Bubela T., Brivanlou A.H., Carpenter M., Charo R.A., Clark A., Clayton E., Cong Y. (2021). Isscr guidelines for stem cell research and clinical translation: the 2021 update. Stem Cell Rep..

[bib92] Magno R., Grieneisen V.A., Marée A.F. (2015). The biophysical nature of cells: potential cell behaviours revealed by analytical and computational studies of cell surface mechanics. BMC Biophys..

[bib93] Maître J.L. (2017). Mechanics of blastocyst morphogenesis. Biol. Cell.

[bib94] Maître J.L., Turlier H., Illukkumbura R., Eismann B., Niwayama R., Nédélec F., Hiiragi T. (2016). Asymmetric division of contractile domains couples cell positioning and fate specification. Nature.

[bib95] Maini P.K. (1999). Mathematics inspired by biology.

[bib96] Maître J.-L., Niwayama R., Turlier H., Nédélec F., Hiiragi T. (2015). Pulsatile cell-autonomous contractility drives compaction in the mouse embryo. Nat. Cell Biol..

[bib97] Manning M.L., Foty R.A., Steinberg M.S., Schoetz E.-M. (2010). Coaction of intercellular adhesion and cortical tension specifies tissue surface tension. Proc. Natl. Acad. Sci. USA.

[bib98] Marée A.F., Grieneisen V.A., Hogeweg P. (2007). Single-cell-based models in biology and medicine.

[bib99] Martin A.C., Goldstein B. (2014). Apical constriction: themes and variations on a cellular mechanism driving morphogenesis. Development.

[bib100] Matthews K.R.W., Iltis A.S., Marquez N.G., Wagner D.S., Robert J.S., de Melo-Martín I., Bigg M., Franklin S., Holm S., Metzler I. (2021). Rethinking human embryo research policies. Hastings Cent. Rep..

[bib101] Mehta R.H. (2014). Sourcing human embryos for embryonic stem cell lines: problems & perspectives. Indian J. Med. Res..

[bib102] Mijailovich S.M., Kojic M., Zivkovic M., Fabry B., Fredberg J.J. (2002). A finite element model of cell deformation during magnetic bead twisting. J. Appl. Physiol..

[bib103] Montes-Olivas S., Marucci L., Homer M. (2019). Mathematical models of organoid cultures. Front. Genet..

[bib104] Morris S.M., Tallquist M.D., Rock C.O., Cooper J.A. (2002). Dual roles for the dab2 adaptor protein in embryonic development and kidney transport. The EMBO Journal.

[bib105] Motosugi N., Bauer T., Polanski Z., Solter D., Hiiragi T. (2005). Polarity of the mouse embryo is established at blastocyst and is not prepatterned. Genes Dev..

[bib106] Moure A., Gomez H. (2020). Dual role of the nucleus in cell migration on planar substrates. Biomech. Model. Mechanobiol..

[bib107] Mueller R., Yeomans J.M., Doostmohammadi A. (2019). Emergence of active nematic behavior in monolayers of isotropic cells. Phys. Rev. Lett..

[bib108] Mutembei H.M., Tsuma V.T., Muasa B., Muraya J., Erastus R. (2015). Bovine in-vitro embryo production and its contribution towards improved food security in Kenya. Afr. J. Food Agric. Nutr. Dev..

[bib109] Najem S., Grant M. (2016). Phase-field model for collective cell migration. Phys. Rev. E.

[bib110] Neilson M.P., Mackenzie J.A., Webb S.D., Insall R.H. (2011). Modeling cell movement and chemotaxis using pseudopod-based feedback. SIAM J. Sci. Comput..

[bib111] Neilson M.P., Veltman D.M., van Haastert P.J.M., Webb S.D., Mackenzie J.A., Insall R.H. (2011). Chemotaxis: a feedback-based computational model robustly predicts multiple aspects of real cell behaviour. PLoS Biol..

[bib112] Newman T.J. (2005). Modeling multi-cellular systems using sub-cellular elements. arXiv.

[bib113] Ni H., Papoian G.A. (2021). Membrane-medyan: simulating deformable vesicles containing complex cytoskeletal networks. J. Phys. Chem. B.

[bib114] Nielsen B.F., Nissen S.B., Sneppen K., Mathiesen J., Trusina A. (2020). Model to link cell shape and polarity with organogenesis. iScience.

[bib115] Nissen S.B., Perera M., Gonzalez J.M., Morgani S.M., Jensen M.H., Sneppen K., Brickman J.M., Trusina A. (2017). Four simple rules that are sufficient to generate the mammalian blastocyst. PLoS Biol..

[bib116] Nissen S.B., Rønhild S., Trusina A., Sneppen K. (2018). Theoretical tool bridging cell polarities with development of robust morphologies. Elife.

[bib117] Okabe A., Boots B., Sugihara K., Chiu S.N. (2009).

[bib118] Osborne J.M., Fletcher A.G., Pitt-Francis J.M., Maini P.K., Gavaghan D.J. (2017). Comparing individual-based approaches to modelling the self-organization of multicellular tissues. PLoS Comput. Biol..

[bib119] Palsson E. (2001). A three-dimensional model of cell movement in multicellular systems. Future Generat. Comput. Syst..

[bib120] Pesce M., Schöler H.R. (2001). Oct-4: gatekeeper in the beginnings of mammalian development. Stem Cells.

[bib121] Plusa B., Piliszek A., Frankenberg S., Artus J., Hadjantonakis A.-K. (2008). Distinct sequential cell behaviours direct primitive endoderm formation in the mouse blastocyst. Development.

[bib122] Ralston A., Rossant J. (2008). Cdx2 acts downstream of cell polarization to cell-autonomously promote trophectoderm fate in the early mouse embryo. Dev. Biol..

[bib123] Ramanathan S.P., Helenius J., Stewart M.P., Cattin C.J., Hyman A.A., Muller D.J. (2015). Cdk1-dependent mitotic enrichment of cortical myosin ii promotes cell rounding against confinement. Nat. Cell Biol..

[bib124] Rayon T., Menchero S., Nieto A., Xenopoulos P., Crespo M., Cockburn K., Cañon S., Sasaki H., Hadjantonakis A.-K., de la Pompa J.L. (2014). Notch and hippo converge on cdx2 to specify the trophectoderm lineage in the mouse blastocyst. Dev. Cell.

[bib125] Rens E.G., Edelstein-Keshet L. (2019). From energy to cellular forces in the cellular Potts model: an algorithmic approach. PLoS Comput. Biol..

[bib126] Revell C., Blumenfeld R., Chalut K.J. (2019). Force-based three-dimensional model predicts mechanical drivers of cell sorting. Proc. Biol. Sci..

[bib127] Ritter M., Bresgen N., Kerschbaum H.H. (2021). From pinocytosis to methuosis—fluid consumption as a risk factor for cell death. Front. Cell Dev. Biol..

[bib128] Rivron N.C., Frias-Aldeguer J., Vrij E.J., Boisset J.-C., Korving J., Vivié J., Truckenmüller R.K., Van Oudenaarden A., Van Blitterswijk C.A., Geijsen N. (2018). Blastocyst-like structures generated solely from stem cells. Nature.

[bib129] Rossant J. (2015). Mouse and human blastocyst-derived stem cells: vive les differences. Development.

[bib130] Rottmann O.J., Lampeter W.W. (1981). Development of early mouse and rabbit embryos without zona pellucida. J. Reprod. Fertil..

[bib131] Salbreux G., Charras G., Paluch E. (2012). Actin cortex mechanics and cellular morphogenesis. Trends Cell Biol..

[bib132] Samarage C.R., White M.D., Álvarez Y.D., Fierro-González J.C., Henon Y., Jesudason E.C., Bissiere S., Fouras A., Plachta N. (2015). Cortical tension allocates the first inner cells of the mammalian embryo. Dev. Cell.

[bib133] Schrode N., Saiz N., Di Talia S., Hadjantonakis A.-K. (2014). Gata6 levels modulate primitive endoderm cell fate choice and timing in the mouse blastocyst. Dev. Cell.

[bib134] Scianna M., Preziosi L. (2012). Multiscale developments of the cellular Potts model. Multiscale Model. Simul..

[bib135] Sego T.J., Aponte-Serrano J.O., Ferrari Gianlupi J., Heaps S.R., Breithaupt K., Brusch L., Crawshaw J., Osborne J.M., Quardokus E.M., Plemper R.K., Glazier J.A. (2020). A modular framework for multiscale, multicellular, spatiotemporal modelling of acute primary viral infection and immune response in epithelial tissues and its application to drug therapy timing and effectiveness. PLoS Comput. Biol..

[bib136] Smyth N., Vatansever H.S., Murray P., Meyer M., Frie C., Paulsson M., Edgar D. (1999). Absence of basement membranes after targeting the lamc1 gene results in embryonic lethality due to failure of endoderm differentiation. J. Cell Biol..

[bib137] Spahn P., Reuter R. (2013). A vertex model of drosophila ventral furrow formation. PLoS One.

[bib138] Stephens L.E., Sutherland A.E., Klimanskaya I.V., Andrieux A., Meneses J., Pedersen R.A., Damsky C.H. (1995). Deletion of beta 1 integrins in mice results in inner cell mass failure and peri-implantation lethality. Genes & development.

[bib139] Tanaka S. (2015). Simulation frameworks for morphogenetic problems. Computation.

[bib140] Tarkowski A.K., Wróblewska J. (1967). Development of blastomeres of mouse eggs isolated at the 4-and 8-cell stage. J. Embryol. Exp. Morphol..

[bib141] Tinevez J.-Y., Schulze U., Salbreux G., Roensch J., Joanny J.-F., Paluch E. (2009). Role of cortical tension in bleb growth. Proc. Natl. Acad. Sci. USA.

[bib142] Tocci A. (2020). The unknown human trophectoderm: implication for biopsy at the blastocyst stage. J. Assist. Reprod. Genet..

[bib143] Trichas G., Smith A.M., White N., Wilkins V., Watanabe T., Moore A., Joyce B., Sugnaseelan J., Rodriguez T.A., Kay D. (2012). Multi-cellular rosettes in the mouse visceral endoderm facilitate the ordered migration of anterior visceral endoderm cells. PLoS Biol..

[bib144] Turlier H., Maître J.L. (2015). Mechanics of tissue compaction. Semin. Cell Dev. Biol..

[bib145] Tvergaard V., Needleman D., Needleman A. (2021). A mechanical model of blastocyst hatching. Extreme Mechanics Letters.

[bib146] Van Roy F., Berx G. (2008). The cell-cell adhesion molecule e-cadherin. Cell. Mol. Life Sci..

[bib147] Vijesh N., Chakrabarti S.K., Sreekumar J. (2013). Modeling of gene regulatory networks: a review. J. Biomed. Sci. Eng..

[bib148] Voss-Böhme A. (2012). Multi-scale modeling in morphogenesis: a critical analysis of the cellular Potts model. PLoS One.

[bib149] Wilder P.J., Kelly D., Brigman K., Peterson C.L., Nowling T., Gao Q.-S., McComb R.D., Capecchi M.R., Rizzino A. (1997). Inactivation of the fgf-4 gene in embryonic stem cells alters the growth and/or the survival of their early differentiated progeny. Dev. Biol..

[bib150] Wildt D.E., Monfort S.L., Donoghue A.M., Johnston L.A., Howard J. (1992). Embryogenesis in conservation biology—or, how to make an endangered species embryo. Theriogenology.

[bib151] Wu G., Schöler H.R. (2014). Role of oct4 in the early embryo development. Cell Regen..

[bib152] Wu N., Pitts M.J. (1999). Development and validation of a finite element model of an apple fruit cell. Postharvest Biol. Technol..

[bib153] Yamanaka Y., Lanner F., Rossant J. (2010). Fgf signal-dependent segregation of primitive endoderm and epiblast in the mouse blastocyst. Development.

[bib154] Yanagida A., Corujo-Simon E., Revell C.K., Sahu P., Stirparo G.G., Aspalter I.M., Winkel A.K., Peters R., De Belly H., Cassani D.A. (2022). Cell surface fluctuations regulate early embryonic lineage sorting. Cell.

[bib155] Yanagida A., Spindlow D., Nichols J., Dattani A., Smith A., Guo G. (2021). Naive stem cell blastocyst model captures human embryo lineage segregation. Cell Stem Cell.

[bib156] Yanez L.Z., Han J., Behr B.B., Pera R.A.R., Camarillo D.B. (2016). Human oocyte developmental potential is predicted by mechanical properties within hours after fertilization. Nat. Commun..

[bib157] Yang D.-H., Smith E.R., Roland I.H., Sheng Z., He J., Martin W.D., Hamilton T.C., Lambeth J.D., Xu X.-X. (2002). Disabled-2 is essential for endodermal cell positioning and structure formation during mouse embryogenesis. Dev. Biol..

[bib158] Yang L., Effler J.C., Kutscher B.L., Sullivan S.E., Robinson D.N., Iglesias P.A. (2008). Modeling cellular deformations using the level set formalism. BMC Syst. Biol..

[bib159] Yu L., Wei Y., Duan J., Schmitz D.A., Sakurai M., Wang L., Wang K., Zhao S., Hon G.C., Wu J. (2021). Blastocyst-like structures generated from human pluripotent stem cells. Nature.

[bib160] Zheng Y., Shao Y., Fu J. (2021). A microfluidics-based stem cell model of early post-implantation human development. Nat. Protoc..

[bib161] Zhu C.-s., Ma F.-l., Lei P., Han D., Feng L. (2021). Comparison between level set and phase field method for simulating bubble movement behavior under electric field. Chin. J. Phys..

[bib162] Zhu M., Shahbazi M., Martin A., Zhang C., Sozen B., Borsos M., Mandelbaum R.S., Paulson R.J., Mole M.A., Esbert M. (2021). Human embryo polarization requires plc signaling to mediate trophectoderm specification. Elife.

